# The sulfur/sulfonates transport systems in *Xanthomonas citri* pv. *citri*

**DOI:** 10.1186/s12864-015-1736-5

**Published:** 2015-07-14

**Authors:** Cristiane Tambascia Pereira, Alexandre Moutran, Melissa Fessel, Andrea Balan

**Affiliations:** Laboratório de Biologia Estrutural Aplicada, Departamento de Microbiologia, Universidade de São Paulo, Av. Prof. Lineu Prestes, 1374, Cidade Universitária, São Paulo, SP CEP 05508-000 Brazil; Laboratório Nacional de Biociências (LNBio), Centro de Pesquisas em Energia e Materiais (CNPEM), Campinas, SP CEP 13083-970 Brazil

**Keywords:** *cys* regulon, *Xanthomonas citri*, Sulfate, Alkanesulfonate transport, ABC transporter

## Abstract

**Background:**

The *Xanthomonas citri* pv. *citri* (*X. citri*) is a phytopathogenic bacterium that infects different species of citrus plants where it causes canker disease. The adaptation to different habitats is related to the ability of the cells to metabolize and to assimilate diverse compounds, including sulfur, an essential element for all organisms. In *Escherichia coli,* the necessary sulfur can be obtained by a set of proteins whose genes belong to the *cys* regulon. Although the *cys* regulon proteins and their importance have been described in many other bacteria, there are no data related to these proteins in *X. citri* or in the *Xanthomonas* genus. The study of the relevance of these systems in these phytopathogenic bacteria that have distinct mechanisms of infection is one essential step toward understanding their physiology. In this work, we used bioinformatics, molecular modeling and transcription analysis (RT-PCR) to identify and characterize the putative *cys* regulon genes in *X. citri.*

**Results:**

We showed that the ATP Binding Cassette Transporter (ABC transporter) SbpCysUWA for sulfate uptake is conserved in *X. citri* and translated in presence of sulfate. On the other hand, differently from what is predicted in databases, according molecular modeling and phylogenetic analysis, *X. citri* does not show a proper taurine transporter, but two different ABC systems related to the alkanesulfonate/sulfonate transport that were recently acquired during evolution. RT-PCR analysis evidenced that these genes and their putative transcriptional regulator CysB are rather transcripted in XAM1, a medium with defined concentration of sulfate, than LB.

**Conclusions:**

The presence of at least three distinct systems for sulfate and sulfonates  assimilation in *X. citri* evidenced the importance of these compounds for the bacterium. The transcription of genes involved with alkanesulfonate/sulfur compounds in XAM1 along to CysB suggests that despite the differences in the transporters, the regulation of these systems might be similar to the described for *E. coli*. Altogether, these results will serve as a foundation for further studies aimed to understanding the relevance of sulfur in growth, virulence and pathogenesis of *X. citri* and related bacteria.

**Electronic supplementary material:**

The online version of this article (doi:10.1186/s12864-015-1736-5) contains supplementary material, which is available to authorized users.

## Background

The plant-associated bacteria from the *Xanthomonas* genus exhibit a high degree of host plant specificity when invading diverse tissues and causing different types of diseases [12]. Comparative genomic analysis has provided insights into the role of horizontal gene transfer and the understanding of the pathogenic adaptations in this genus [40]. *X. citri* is one of the relevant species from the genus because it is the causative agent of the citrus canker, a disease that affects citrus plants and causes significant economic losses in Brazil and many other countries in the world. Although studies have demonstrated the importance of specific genes for biofilm formation [23], infection [20] and pathogenesis [30], there is a lack of information regarding the sulfur or sulfate assimilation pathways in this bacterium and the relevance of these compounds for infection and pathogenesis.

In *Escherichia coli*, the *cys* regulon genes encode a set of proteins that are associated with the acquisition of sulfate and organosulfur compounds, such as sulfonates (R-SO3-) and sulfate esters (RO-SO3-), as sulfur source for cysteine biosynthesis. The preferable source of sulfur is sulfate, which is transported by the ABC transporter SbpCysAUW. Once the sulfate assimilation is completed, CysNCDH proteins reduce it to sulfite, and the CysGIJ complex reduces sulfite to sulfide [14]. In sulfate or cysteine starvation, the operons *ssuABCDE* and *tauABCD* are induced to constitute two ABC transporters that are required for uptake of alkanesulfonate (SsuABC) and taurine (TauABC), respectively, as well as the enzymes for the desulfonation of the organosulfonates (SsuDE and TauD, respectively) [9, 38]. The regulation of these genes is mediated by Cbl and CysB proteins, which consist of two LysR-type transcriptional activators [31, 37, 39]. CysB is the regulator for sulfur assimilation in *E. coli*, while the Cbl protein functions as an accessory element that is specific for the utilization of sulfur from organosulfur sources [39], activating the expression of the *tau* and *ssu* genes. Both regulators are closely related, sharing 41 % sequence identity and 60 % similarity [31].

The presence and importance of these systems have been shown for several bacterial species such as *Salmonella typhimurium* [35], *Pseudomonas aeruginosa* [13], *Bacillus subtilis* [11], and *Acidithiobacillus ferrooxidans* [36]. In addition, sulfated metabolites have been implicated in the interactions between bacteria and their eukaryotic hosts, including species of the plant symbiont genus *Rhizobium* [5], *Mycobacterium tuberculosis* [16] and *Xanthomonas oryzae* [6]. Recently, our group has expressed, purified and solved the three-dimensional structure of the alkanesulfonate-binding protein SsuA bound to three different alkanesulfonates. Through the monitoring of growth, infection and pathogenesis in *Citrus sinensis* leaves, we showed the importance of an alkanesulfonate binding protein for the growth, infection and production of xantham gum and the development of the canker citric phenotype [2].

Based on the previous information that has been described for *E. coli* and other microorganisms, we carried out bioinformatics and trancriptional analyses (RT-PCR) of *X. citri* to identify the putative *cys* regulon components. The genes belonging to the *cys* regulon in *E. coli* were used as template for a BlastP search against to *X. citri* genome. The protein sequences were compared and modeled, and the putative motifs and domains were characterized. Moreover, the transcription of genes belonging to the ABC transporters and some genes from *cys* pathway and *ssu* operons was evidenced by RT-PCR, suggesting they are required for the bacterial growth. Using this information, we were able to develop a model for sulfur assimilation in *X. citri* evidencing the expression of genes of the sulfate uptake and sulfur assimilation pathway. Moreover, the data show that *X. citri* presents significant differences related to the systems for uptake of aliphatic sulfonates and alkanesulfonates, as well as the ability to use sulfate- and sulfur-reduced compounds as sources of energy.

## Methods

### Search for *cys* regulon gene orthologs, sequence alignment and phylogenetic analysis

The genes belonging to the *cys* regulon in *E. coli*, as previously described [9, 14, 37–39], were obtained from the KEGG2 server (Bioinformatics Center Institute for Chemical Research Kyoto University, www.genome.jp) and used to perform a basic local alignment search BlastN (http://blast.ncbi.nlm.nih.gov) against the *X. citri* (TaxId: 346) genome database (Additional file 1: Table A1). All the default parameters of the program were used. The amino acid sequence alignments were carried out using ClustalX [34] and edited with GeneDoc [18]. To build the phylogenetic tree of the periplasmic components from the ABC transporters related to sulfate or sulfonates uptake found in *X. citri*, the gene sequences of the periplasmic proteins [*ssuA1* (GeneID: 1154920), *ssuA2* (GeneID: 1157269) and *sbp* (GeneID: 1155088)] were submitted to the BlastP using the non-redundant sequence database. The criterion of choice of the proteins and microorganisms was based on the diversity of genus and similarity of function of the proteins. Description of the hosts and sequences is shown in Additional file 2: Table A2. Phylogenetic reconstruction and molecular evolutionary analyses were conducted with MEGA version 5 [33], using the neighbor-joining statistical method, *p*-distance to estimate the evolutionary distances, and 1000 Bootstrap Replications [24]. All the gaps were treated as a complete deletion. The tree that shows the conservation of the *cys* regulon genes in different phylogenetic groups was built based on the 16S rDNA sequences from the microorganisms described in Additional file 3: Table A3. All the organisms with complete genome sequences were named in agreement with the codes of the KEGG Organisms Complete Genomes table. To name the microorganisms whose genomes were incomplete or absent, we used the capital first letter of the genus followed by two or three first letters from the species.

### Molecular modeling

Molecular modeling of the proteins SsuA1, SflA and SsuD1 was performed using the Modeller 9v4 program [25] with basic (for one template) or advanced (for more than one template) scripts (http://salilab.org/modeller/tutorial/). A total of 10 models were generated for each target protein, and the best model was selected using the lowest value of the objective function curve. The templates used for the model building of each protein, as well as the Protein Data Bank (PDB) code and amino acid sequence identities, are presented in Additional file 4: Table A4. Consensus prediction of transmembrane domains was obtained with TOPCONS [3].

### RNA extraction, cDNA synthesis and RT-PCR

The *X. citri* strain used in this study was grown at 28 °C overnight in Luria-Bertani (LB) modified broth (without NaCl) supplemented with ampicillin (100 μg/ml) at 28 °C at 200 rpm. After the growth period, the cultures were diluted 50 times, washed two times in sterile water and incubated in virulence induction medium to mimic the plant environment, XAM1 [10, 30], or LB until the mid end-exponential growth phase. Samples normalized by O.D. to contain about 10^14^ cells of each culture were transferred to a microcentrifuge tube and centrifuged for 2 min at 14,000 × *g*. We carefully remove the supernatant, leaving the pellet as dry as possible for suspension in 100 μl freshly prepared TE buffer containing lysozyme (10 mg/ml). The mixture was incubated at room temperature for 5 min. The following steps were performed according to the SV Total RNA Isolation System protocol (Promega, Madison, MA, USA). To check that there wasn’t DNA contamination we performed PCRs using the RNA samples and no amplifications were detected.

Reverse transcription was carried out on the day after RNA isolation using the GoScript™ Reverse Transcription System (Promega, Madison, MA, USA). A total mix of 50 ng RNA and 0.5 μg Random Primer per reaction was added, and the final volume was brought up to 5 μl with nuclease-free water. The RNA-primer mix was heated at 70 °C for 5 min followed by chilling in ice water for 5 min. Then, the RNA-primer mix was added to the reverse transcription reaction mix. For each cDNA reaction, the reverse transcription reaction mix was composed of the following: 4 μl GoScript™ 5x Reaction Buffer, 2 μl MgCl_2_, 1 μl PCR Nucleotide Mix, 1 μl GoScript™ Reverse Transcriptase and 7 μl nuclease-free water to a final volume of 15 μl. The RT-PCR temperature sequence was as follows: 25 °C for 5 min to assist annealing, incubation at 42 °C for one hour and incubation at 70 °C for 15 min to inactivate the reverse transcriptase. The cDNA was stored at -20 °C. The PCR was carried out in a 25 μl reaction mixture, using 1 μl of the RT (200 ng template for cDNAs and 100 ng for genomic DNA) reaction as template for 0,5 μl Taq DNA (5U/μl) and 20 pmol of each primer (Additional file 5: Table A5). As positive control of the reactions we used specific oligonucleotides to amplify a 271 bp 16S ribosomal RNA fragment (KEGG number XAC3896). The temperature of annealing for all genes was 51 °C. Amplification was performed in 40 cycles with a T1000 Thermo Cycler (BIO-RAD, Philadelphia, USA).

## Results and discussion

### *X. citri* conserves the Cys proteins for sulfate transport and desulfonation to sulfide

**Fig. 1:**
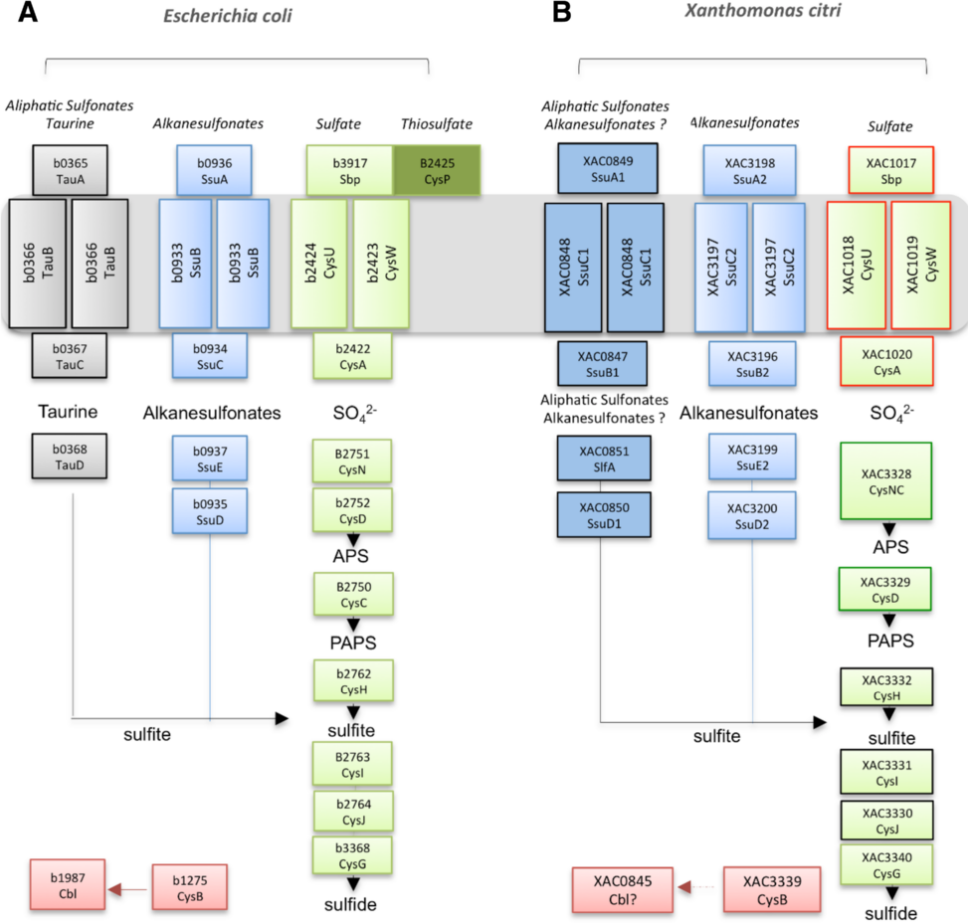
Overall scheme of the predicted *cys* regulon genes from *Escherichia coli* and putative orthologs identified in *X. citri* genome. (**a**) *cys* regulon genes from *E. coli* and (**b**) orthologs found in *X. citri*. Green boxes indicate the genes for the sulfate (SO_4_^2-^) uptake system that are responsible for binding and reduction to sulfide, through a set of reactions whose intermediates are adenosine-5’-phosphosulfate (APS), 3’-phosphoadenosine-5’-phosphosulfate (PAPS) and sulfite. Identical colored lines (red, green and black) in boxes from *X. citri* genes represent genes that belong to the same predicted operon. CysP, only identified in *E. coli*, is the periplasmic component for thiosulfate uptake that uses the same sulfate transporter. Light blue boxes indicate the genes that are described for the transport and assimilation of alkanesulfonates. Based on the sequence identity and molecular modeling, the taurine transporter and TauD of the *E. coli* pathway (shown in gray) were not identified in *X. citri*, but seem to be replaced by other homologs of putative alkanesulfonate or aliphatic sulfonate transporter and desulfonative enzymes (SsuD1 and SlfA) (darker blue boxes). The red boxes show the regulatory genes, *cysB*, *cbl*. All genes are identified by their KEGG code and when present, the name of the putative protein. P: periplasmic space, I.M.: inner membrane, C: cytoplasm

*E. coli* contains at least 27 genes encoding the proteins that belong to the *cys* regulon [14, 37]. The search for orthologs of these genes in the *X. citri* genome revealed that the phytopathogenic bacterium conserves the ABC transporter SbpCysUWA and almost all the enzymes for sulfate to sulfide reduction, as shown in Fig. 1 (green boxes in a and b). All these proteins in *X. citri* are supposely encoded by the same predicted operon (Fig. 1, red lines in the boxes) and shared high amino acid sequence identity when compared to the *E. coli* orthologs (47 % to 66 %) (Additional file 1: Table A1). *X. citri* Sbp shows conservation of 100 % of the residues that are involved with sulfate binding. According to prediction of transmembrane segments using TOPCONS server [3], the permeases CysU/CysW form a heterodimer that consist of 12 transmembrane spanning helices (6 from each protein). All the proteins putatively involved with sulfate reduction were also identified and conserved in *X. citri*. Two genes, *nodQ* (Xac3328) and *cysD* (Xac3329)*,* encode respectively, a fusion protein that consists of the sulfate adenylyltransferase subunit 1/adenylylsulfate kinase or CysNC, as previously has been identified in other microorganisms such as *Pseudomonas syringae* [17] and *Rhizobium meliloti* [26], and the subunit 2 of the sulfate adenylyltransferase (Additional file 1: Table A1)*.* In *Rhizobium meliloti* the NodQ protein that shares 58 % of amino acid sequence identity with the related protein in *X. citri,* is involved in the synthesis of nodulation factors that are active on the roots of alfalfa as well as in the formation of activated sulfate intermediates, which will be transferred to the nodulation factors by NodH [26, 27]. CysH (Xac3332) in *X. citri* rather works as a PAPS reductase since it exhibits the conserved (KRT)ECG(LS)H signature of the APS/PAPS reductase family, but not the critical four cysteines that coordinate a 4Fe-4S center in APS reductases and it conserves the C239 that is responsible for dimerization in PAPS reductases, according to Kopriva and collaborators [15]. The same operon encodes CysI and CysJ, which exhibit respectively, the conserved domains of ferredoxin-like nitrite/sulfite reductase (PF03460), nitrite and sulfite reductase 4Fe-4S (PF01077), and the cysteines that are responsible for iron-sulfur coordination (Additional file 6: Figure A1-A). Searching for CysG, a syroheme synthase, we found 3 distinct predicted *cysG* genes located in two different positions in the genome (Additional file 7: Figure A2-A). The three proteins shared low sequence identity among them (15-23 %) but the BlastP against the protein data bank resulted in the same three-dimensional structure of the syroheme synthase CysG from *S. typhimurium* (PDB code 1PJQ) [32]. The comparison among the domains of *S. typhimurium* CysG and the three CysG from *X. citri,* showed that only Xac3340 presented the necessary motifs for the predicted activity (Additional file 7: Figure A2-B). Although Xac3340 encodes an enzyme that fits better in the model of a siroheme synthase, the presence of two other genes with related functions arise the possibility that in *X. citri* the synthesis of siroheme be mediated by more than one enzyme. In fact, the use of three or two enzymes for siroheme synthesis has been described before for *Bacillus megaterium* [22] and *Saccharomyces cerevisiae* [29], respectively. These data show that the synthesis of syroheme is not clearly defined in *X. citri* and additional functional studies need to be done.

### Systems for the transport of alkanesulfonate and aliphatic compounds in *X. citri*

**Fig. 2:**
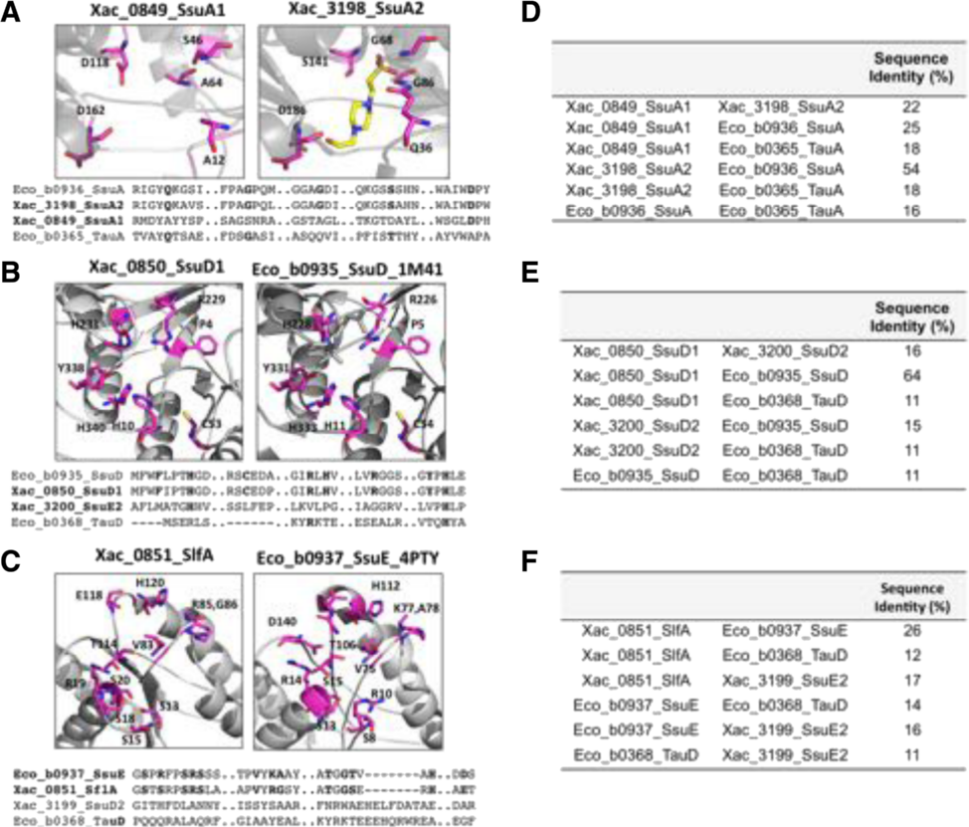
Molecular modeling, structural comparison and amino acid sequence conservation of the proteins encoded by *ssu1* and *ssu2* predicted operons. Structural comparisons are shown for (**a**) *X. citri* SsuA1 (Xac0849) and the alkanesulfonate-binding protein SsuA2 [2]; (**b**) SsuD1 (Xac0850) with the crystal structure of the *E. coli* monooxygenase SsuD [8]; and (**c**) SlfA (Xac0851) with the crystal structure of the *E. coli* alkanesulfonate FMN reductase SsuE [7]. Protein structures are shown in gray cartoon and the putative residues involved in the substrate binding in magenta stick. Amino acid sequence alignment of the pocket regions are shown below the structures including the related proteins for each group with residues of the active site shown in bold. Amino acid sequence identity of the periplasmic-binding proteins, monooxygenases and oxidoreductases are respectively compared in the tables (**d**), (**e**) and (**f**). All the proteins used for sequence analysis, structural comparison and molecular modeling are described in Additional file 1: Table A1 and Additional file 4: Table A4 from Additional files. Sequence alignments were performed with ClustalX [34]

In *E. coli,* during sulfate starvation or depletion, two other operons are induced for the transport and desulfonation of alkanesulfonates and taurine, respectively *ssuABCDE* and *tauABCD* [9, 37, 38]. The organizational scheme of these transporters is evidenced in Fig. 1a (blue and gray, respectively) and shows that besides the transporter, the predicted operons encode the TauD (*tauABCD* operon) and the two-component system SsuD and SsuE (*ssu* operon) for the desulfonation of the organosulfonates such as taurine and alkanesulfonates, respectively. In contrast, the *X. citri* genome search for these genes resulted in the identification of two distinct predicted operons here denominated *ssu1* (*slfAssuDACB*) and *ssu2* (*ssuDEACB*) (Fig. 1, dark and clear blue, respectively). We previously characterized the three-dimensional structure of the *X. citri* SsuA2 (Xac3198) in presence of alkanesulfonates and showed its importance for bacterial growth in minimal media when alkanesulfonates were the unique source of sulfur. Indeed, *Citrus sinensis* leaves infected with the mutant strain demonstrated an attenuated symptom of the canker disease, suggesting a role of this protein in the infection and pathogenesis mechanisms [2]. A structural comparison and amino acid sequence alignment show that *X. citri* SsuA2 shares 54 % of amino acid sequence identity with *E. coli* SsuA2 and conserves all the residues that interact with the alkanesulfonate (Fig. 2a alignment and d). In addition, the predicted operon *ssu2* also has genes encoding the ABC transporter, SsuC2 (permease) and SsuB2 (ATPase), and the putative two-component system for alkanesulfonate reduction, composed of the nitriloacetate monooxygenase SsuD2 (Xac3200) and the oxidoreductase SsuE2 (Xac3199). However, *X. citri* SsuD2 and SsuE2 do not show amino acid sequence identity with the previous studied orthologues from *E. coli*, or even with the putative proteins encoded by operon *ssu1* (Xac0850 and Xac0851) (Fig. 2e and f, respectively). This fact arises questions about the functionality of these proteins or the two-component system or might show differences in their mechanisms of action. Interestingly, the predicted operon *ssu1* encodes an additional transport system that is distinct from those present in *X. citri* and *E. coli*. To get structural information related to active site, residues for interaction and possible similarities among these proteins and those from Ssu2 system, we modelled SsuA1, SlfA and SsuD. SsuA1 model was built based on the structural coordinates from *X. citri* SsuA2 (22 % of amino acid sequence identity) [2] but revealed significant differences in the ligand-binding pocket (Fig. 2a), suggesting that the protein could interact with different range of ligands. Conversely, the structural models of SsuD1 and SlfA showed higher sequence identity with the alkanesulfonate monooxygenase SsuD (b0935) [8] (Fig. 2b and e) and the NAD(P)H-dependent FMN reductase SsuE (b0937) [7] from *E. coli* (Fig. 2c and f), orthologues used as templates for the structural models. The data obtained from proteins encoded by the putative *ssu1* operon suggest its role as a second alkanesulfonate transporter system.

### Phylogenetic analysis

**Fig. 3:**
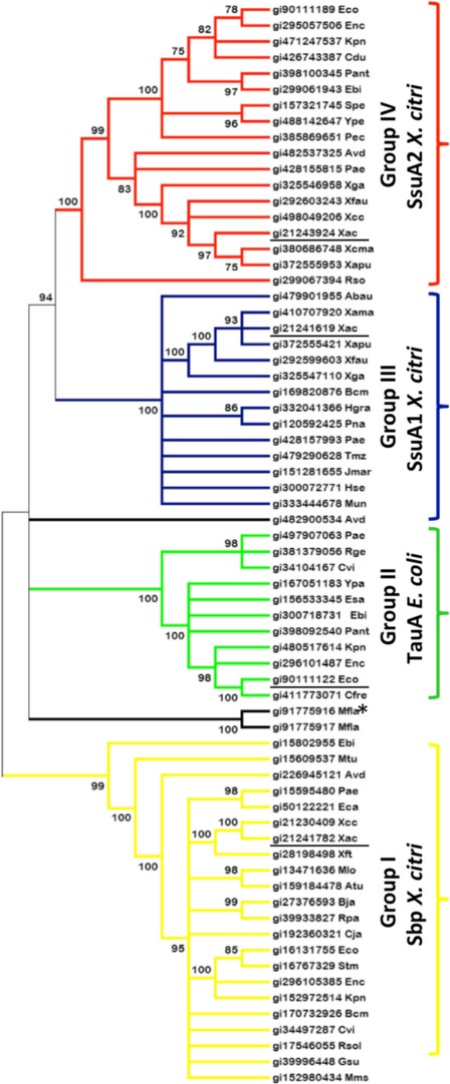
Phylogenetic analysis of the *X. citri* Sbp, SsuA1 and SsuA2 and their relationship with orthologs identified in different microorganisms. The list of proteins and their reference codes at NCBI are described in Additional file 2: Table A2. Proteins were classified essentially in four groups as the following: Group I (yellow) for sulfate and thiosulfate-binding proteins (Sbp and CysP); Group II (green) for taurine-binding proteins (TauA); Group III (blue) for alkanesulfonate and sulfate ester binding proteins that show higher sequence similarity with the ortholog of *X. citri* SsuA1; and Group IV (red) for alkanesulfonate binding proteins, which best represented by SsuA from *E. coli* and SsuA from *X. citri.* The putative sulfonate-binding protein from *Methylobacillus flagellates* KT, which had its three-dimensional structure solved (PDB code 3UIF), was classified in a separate branch (black lines with asterisk) together with its paralog. The tree was generated with the Neighbor-Joining Method (1000 bootstrap) using the MEGA program version 5 [33]. *X. citri* proteins and *E. coli* TauA were underlined in each group, as a reference

**Fig. 4:**
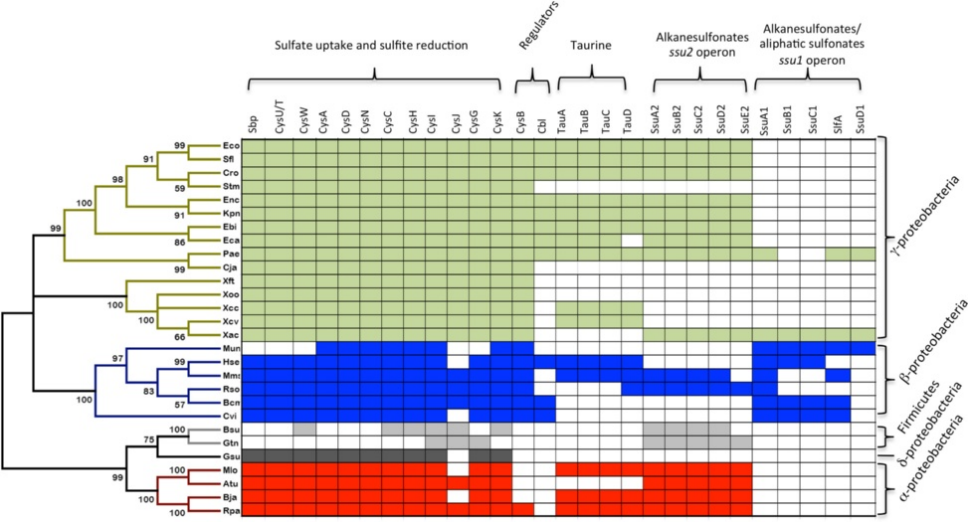
Conservation of *cys* regulon proteins. A relationship between the evolution of bacteria and the presence of the *cys* regulon genes is shown. The list of microorganisms and 16S rRNA sequences used to produce the phylogenetic tree is shown in Additional file 3: Table A3. Termicutes, Chlamydia and Spyrochaetae phyla are not represented because they did not show any evidence of containing orthologs of the *cys* regulon genes. The microorganisms are represented as a three letters code according to KEGG. All the *cys* regulon genes are shown as horizontal bars in the table. Colored boxes show the presence of one gene in a determined species. Boxes with an asterisk (*) inside represent fusioned genes. The phylogenetic tree was generated with MEGA version 5 [33] using the Neighbor-Joining Method (1000 bootstrap)

The previous analyses suggested that *X. citri* had two ABC systems for the transport and assimilation of organosulfur compounds, but it lacks a proper TauABCD system as observed in *E. coli.* In addition, the bioinformatics analysis of the *X. citri* and *E. coli* components based on sequence alignment revealed significant differences among the periplasmic components, suggesting diversity in substrate affinity and specificity. To compare the four different periplasmic-binding proteins, we coupled phylogenetic analysis using a group of orthologs (Fig. 3) and the structural information obtained from molecular modeling. As previously demonstrated [4, 21], Spb proteins (Group I in yellow) form a separate group from the alkanesulfonate and aliphatic sulfonate binding proteins. Interestingly, the other proteins were classified in three different groups: Group II (green) for the orthologs of *E. coli* taurine-binding protein TauA, Group III (blue) for the orthologs of *X. citri* SsuA1 (Xac0849) and Group IV (red) for the orthologs of *X. citri* SsuA2. *X. citri* SsuA1 does not belong to the branch of SsuA2, which represents the alkanesulfonate-binding proteins, or the TauA group for taurine-binding proteins, but it belongs to an intermediary group between both proteins. In fact, these data are endorsed by the significant differences found in the *X. citri* SsuA1 ligand-binding pocket when compared to SsuA2 and *E. coli* TauA (Fig. 2d). To verify if orthologues of the proteins of *X. citri* involved in the pathways of sulfate and putative organosulfur compounds were conserved during the evolution, we chose microrganisms from distinct classes to built a phylogenetic tree based on the 16S rRNA sequences (Additional file 3: Table A3). The results show that the system for sulfate uptake and assimilation is highly conserved in the proteobacteria (Fig. 4, colored boxes). The tree suggested a recent evolution of the genes belonging to the alkanesulfonates and organosulfur compounds pathways. Indeed, putative orthologs of the *ssu1* predicted operon are only present in beta-proteobacteria, with exception evidenced for *P. aeruginosa,* which may suggest a possible horizontal gene transfer between this bacterium and *X. citri.* The oldest ancestor found was the *tauA* from *Thermotoga maritima* (tma), a hyperthermophilic bacterium. Ancestral proteins were likely preferably able to bind different compounds than the sulfonates that are transported by the *E. coli* SsuA and TauA proteins, which include the diversity of alkanesulfonates as MOPS, HEPES, ethanesulfonates, propanesulfonates, hexanesulfonates, octanesulfonates and decanesulfonates [9]. The presence of these genes reveals an adaptation to new and competitive environments. Interestingly, orthologues from the *ssu1* and *ssu2* predicted operons were not found in *Xanthomonas campestris* pv. *campestris* (Xcc), *Xanthomonas campestris* pv. *vesicatoria* and *Xylella fastidiosa Temecula* 1 (Fig. 4). *Xyllela*, a xylem-limited bacterium that is insect-vectored to a variety of hosts [28] presented only the *sbpcysWUA* predicted operon, while in *X. campestris* and *X. vesicatoria*, an evolutionarily older group of *tau* operon has been identified. This predicted operon has an evolutionary divergence from other operons, which gave rise to the putative *ssu* operon (Fig. 4). An interesting feature of this phylogenetic tree is the evidence of the importance of the sulfur uptake systems for *X. citri*, a species that can survive in soils where the availability of sulfate is limited and where most sulfur sources originate from organosulfur compounds [9].

### Gene expression of the components from *cys* regulon and predicted *ssu* operons in *X. citri*

**Fig. 5:**
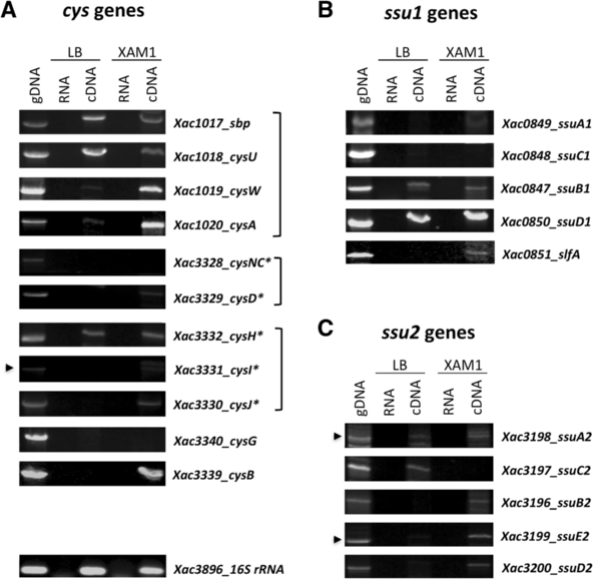
RT-PCR analysis of the gene expression from the predicted *cys* pathway, *ssu1* and *ssu2* operons of *X. citri*. (**a**) Genes from the predicted *cys* regulon; (**b**) genes from predicted operons *ssu1* and (**c**) *ssu2*. Total RNA was extracted from cells grown in LB and XAM1 media during mid end-exponential growth phase. RT-PCR was carried out as described in the Methods. gDNA: genomic DNA (100 ng); cDNA: complementary DNA (200 ng); RNA: negative control. Genes are described according the number followed by the correspondent name as shown in KEGG data bank. Proteins encoded by the genes showed with asterisk (*) had the expression identified in the proteomic analysis of *X. citri* extracts obtained after growth in XAM1 medium [10]. Blankets group genes that belong to the same predicted operons. As a positive control a 271 bp fragment of the 16S rRNA is shown. The arrows indicate the expected molecular mass for each transcript

To verify if the putative genes involved with sulfate or organic sources of sulfate uptake and assimilation were expressed during the bacterium growth in two defined conditions, we performed a RT-PCR analysis from samples obtained from rich medium LB modified and virulence inducing medium XAM1, which final sulfate concentration is 7.8 mM [10, 30]. Total RNA samples were extracted from the *X. citri* cells grown in the different culture media and used for cDNA amplification (Fig. 5). The results showed that transcripts from the ABC transporter *sbpcysUWA* were amplified with expected molecular mass from cells cultured in LB modified and XAM1 media (Fig. 5a), indicating that this transport system is expressed in both conditions. In accordance with these results, the periplasmic component Sbp was identified in proteomic analysis of *X. citri* extracts obtained after growth of the cells in LB and the A minimum medium [19]. Curiously, with exception of the *cysH* none of the other genes related to the sulfur pathway were evidenced in LB modified. On the other hand, *cysD*, *cysI* and *cysJ* were observed in XAM1, corroborating previous results based on proteomics analysis from extracts of *X. citri* after growth in XAM1 medium [10]. Since *cysD* and *cysJ* belong to predicted operons that also encode *cysNC* and *cysHI*, respectively, it is possible that the specific conditions used for amplification or amount of transcripts were not suitable for these genes. The gene that encodes the putative CysG (Xac3340) was not identified. Similarly, we tried to amplify the additional *cysG* genes without success. Still in XAM1, we observed the amplification of *cysB* that shares 39 % of amino acid sequence identity with the putative *E. coli* orthologue. In *E. coli, cysB* encodes a LysR-type transcriptional activator of the sulfate starvation induced genes needed for activation of the *ssu* and *tau* operons in low levels of sulfate [39]. In accordance with the amplification of *cysB* in XAM1, all *ssu* genes (operons 1 and 2), exception of *ssuC2,* also were amplified from cDNA obtained from this medium (Fig. 5b and c). This result is in accordance with the identification of SsuD2 in extracts of *X. citri* obtained after growing in XAM1 [10], and the demonstration that *X. citri* has the capability to grow in minimum medium M9 when the only source of sulfur was alkanesulfonates [2]. If the regulation of the systems found in *X. citri* is related to that of *E. coli,* the presence of Ssu proteins should be evidenced only in very low levels or absence of sulfate, which would be obtained only after log phasis, explaining the absence of the proteins belonging to the transporter systems and the low level of amplification. Intringly, *ssuB1, ssuD1, ssuA2* and *ssuC2* also were amplified from cDNAs obtained of cells cultivated in LB modified. Since samples were obtained from this and XAM1 media during mid end-exponential growth phase, it is expected that sulfate levels be reduced when compared to the initial of growth and still different between both conditions. A detailed analysis of the physiological conditions that induce the expression of these proteins present in distinct species of the *Xanthomonas* genus is an exciting field that can reveals the importance of sulfur transport systems during the infection and patogenesis in these bacteria.

## Conclusions

In this work, we showed for the first time, that the phytopatogenic bacterium *X. citri* has the ABC transporter for sulfate and two additional systems for the transport and oxidoreduction of alkanesulfonates or organosulfur compounds, respectively, SlfASsuDACB (Ssu1) and SsuDEACB (Ssu2). These systems were differentially induced in LB and XAM1. Comparative and phylogenetic analysis of the periplasmic binding proteins, as well the complete set of genes, showed that SlfASsuDACB differs from the classical taurine transporter and probably was originated from the β-proteobacteria group. The presence of more than one transporter for this kind of compound would give to the *X. citri* the advantageous capability to transport and assimilate distinct sources of sulfur, which is relevant for the bacterium maintenance and growth. The absence of these transporters in *X. campestris*, which has a different way of infection and pathogenesis, may also reflect the importance of these compounds for the bacterium. Since there is no previous data related to the sulfur assimilation in *X. citri* or *Xanthomonas* genus, the work presented here is an important step for understanding global sulfur metabolism in these bacteria and arises perspectives for further experimental investigations.

## Additional files

Additional file 1: Table A1. List of putative orthologues of *cys* regulon genes as described for *Escherichia coli* [10, 15, 38–40] found in the *X. citri* genome. Sequences of the *E. coli* genes were used for BlastN against the *Xanthomonas axonopodis* pv. *citri* 306 genome. Nucleotide sequence identities were calculated after the pairwise sequence alignment using the EMBOSS Water program (http://www.ebi.ac.uk/Tools/psa/emboss_water/nucleotide.html). Additional file 2: Table A2. List of proteins and their hosts used for the phylogenetic analyses of the periplasmic components from *X. citri* belonging to the putatives sulfate and organosulfur compounds pathways. Additional file 3: Table A3. Microorganisms and access code for the 16S rRNA sequences used to show the conservation of the putatives sulfate and organosulfur compounds pathways proteins in different classes. Additional file 4: Table A4. Proteins with three-dimentional structure solved used as template for the molecular modeling of the putative sulfur pathway components identified in *X. citri.* Amino acid sequence alignments were performed using ClustalW [35]. Additional file 5: Table A5. Oligonucleotides used for gene amplification in the RT-PCR analysis performed in this study. F: forward and R: reverse oligonucleotides. PCR reactions were described in Methods, using the annealing temperature of 51 °C for all reactions. Additional file 6: Figure A1. Domain structure of the putative CysI and CysJ of *X. citri*. (A) PFAM domain structure of the putative CysI of *X. citri*. PF03460 = nitrite/sulfite reductase ferrodoxin-like half domains and PF01077 = nitrite and sulfite reductase 4Fe-4S binding and siroheme binding. The amino acid sequence (residue numbers 468 to 484) of the PF01077 domain corresponding to the C-terminus of the 4Fe-4S binding and siroheme binding domain is aligned with similar regions of CysI from *Escherichia coli* (KEGG entry: b2763) and *Mycobacterium tuberculosis* H37Rv (gi: 15609528). Conserved amino acids are underlined bold. The sequence of the Prosite motif PS00365, [STV]-G-C-x(3)-C-x(6)-[DE]-[LIVMF]-[GAT]-LIVMF] is also shown. (B) PFAM domain structure of the putative CysJ of *X. citri.* PF00258 = flavodoxin domain, PF00667 = FAD binding domain and PF00175 = NAD-binding domain. The amino acid sequence (residue numbers 317 to 442) of the PF00175 domain is aligned with similar regions of CysJ from *Escherichia coli* (KEGG entry: b2764). Additional file 7: Figure A2. Comparison of the three putative CysG protein sequences found in *X. citri* with the siroheme synthase CysG from *S. typhimurium.* (A) Positioning of the *cysG* genes in the *X. citri* genome. Xac2159 and Xac2157 genes, encoding respectively, an uroporphyrin-III C-methyl transferase and a syroheme synthase, are closely located in opposite directions. The third gene, Xac3341, encoding another syroheme synthase is located in the same predicted operon with the *cysK* gene that encodes a cysteine synthase. (B) Putative conserved domains identified in the three CysG amino acid sequences in comparison with the multifunctional methyltransferase/dehydrogenase/ferrochelatase CysG from *S. typhimurium,* which three-dimensional structure was solved in presence of S-adenosyl-L-homocysteine (SAH) [1]. The amino acid sequence alignment from the identified domains is shown evidencing the conservation of the residues involved with SAH and NAD interactions (showed in bold).
