# Supplementary material for: The sulfur/sulfonates transport systems in Xanthomonas citri pv. citri
Source: BMC Genomics. 2015 Jul 14;16(1):524. doi: 10.1186/s12864-015-1736-5 (PMC4501297; doi:10.1186/s12864-015-1736-5)
Supplement: Additional file 1: Table A1. — List of putative orthologues of cys regulon genes as described for Escherichia coli [10, 15, 38–40] found in the X. citri genome. Sequences of the E. coli genes were used for BlastN against the Xanthomonas axonopodis pv. citri 306 genome. Nucleotide sequence identities were calculated after the pairwise sequence alignment using the EMBOSS Water program (http://www.ebi.ac.uk/Tools/psa/emboss_water/nucleotide.html). [file 12864_2015_1736_MOESM1_ESM.pdf]

Additional File 1

Gene name in *E. coli*	Kegg Entry	Kegg Function Definition	Sequence Identity (%)	Gene name in *X. citri*	Kegg Entry	Kegg Function Definition
sbp	b3917	sulfate transporter subunit	66	*sbp*	XAC1017	ABC transporter sulfate-binding protein
cysU	b2424	sulfate/thiosulfate ABC transporter permease	50	*cysU*	XAC1018	ABC transporter sulfate permease
cysW	b2423	sulfate transport system permease protein	47	*cysW*	XAC1019	ABC transporter sulfate permease
cysA	b2422	sulfate/thiosulfate transporter subunit	56	*cysA*	XAC1020	sulfate ABC transporter ATP-binding protein
cysN	b2751	sulfate adenylyltransferase, subunit 1	52	*nodQ(cysN)*	XAC3328	bifunctional sulfate adenylyltransferase subunit 1
cysC	b2750	adenosine 5'-phosphosulfate kinase	60	*nodQ(cysC)*	XAC3328	adenylylsulfate kinase
cysD	b2752	sulfate adenylyltransferase subunit 2	67	*cysD*	XAC3329	sulfate adenylyltransferase subunit 2
cysH	b2762	3'-phosphoadenosine 5'-phosphosulfate reductase	64	*cysH*	XAC3332	phosphoadenosine phosphosulfate reductase
cysI	b2763	sulfite reductase<br>NAD(P)-binding, heme-binding	50	*cysI*	XAC3331	sulfite reductase subunit beta
cysJ	b2764	sulfite reductase	42	*cysJ*	XAC3330	NADPH-sulfite reductase flavoprotein subunit
cysG	b3368	III dehydrogenase and siroheme ferrochelatase/<br>uroporphyrinogen methyltransferase	54	*cysG*	XAC3340	uroporphyrin-III C-methyltransferase / precorrin-2<br>dehydrogenase / sirohydrochlorin ferrochelatase
cysB	b1275	DNA-binding transcriptional dual regulator,<br>O-acetyl-L-serine-binding	39	*cysB*	XAC3339	transcriptional regulator CysB-like protein
				flbD	XAC0845	transcriptional regulator
cysK	b2414	cysteine synthase A,<br>O-acetylserine sulfhydrolase A subunit	47	*cysK*	XAC3341	cysteine synthase
ssuA	b0936	putative aliphatic sulfonate binding protein	58	*ssuA1*	XAC0849	sulfonate-binding protein
				ssuA2	XAC3198	alkanesulfonate transporter substrate-binding subunit
ssuB	b0933	sulfonate transport system ATP-binding protein	59	*ssuB1*	XAC0847	ABC transporter ATP-binding protein
				ssuB2	XAC3196	ABC transporter ATP-binding subunit
ssuC	b0934	sulfonate transport system permease protein	72	*ssuC1*	XAC0848	ABC transporter permease
				ssuC2	XAC3197	ABC transporter permease
ssuD	b0935	alkanesulfonate monooxygenase, FMNH(2)-dependent	65	*ssuD1*	XAC0850	alkanesulfonate monooxygenase
				ssuD2	XAC3200	nitrilotriacetate monooxygenase
ssuE	b0937	NAD(P)H-dependent FMN reductase	31	*ssuE1*	XAC0851	NADH-dependent FMN reductase
				ssuE2	XAC3199	oxidoreductase
tauA	b0365	taurine transport system substrate-binding protein	25	*ssuA2*	XAC3198	alkanesulfonate transporter substrate-binding subunit
tauB	b0366	taurine transport system ATP-binding protein	44	*nrtCC*	XAC0828	ABC transporter ATP-binding component
tauC	b0367	taurine transport system permease protein	36	*ssuC1*	XAC0848	ABC transporter permease
tauD	b0368	taurine dioxygenase, 2-oxoglutarate-dependent	39	*tauD*	XAC0830	Taurine dioxigenase
