# Supplementary material for: The sulfur/sulfonates transport systems in Xanthomonas citri pv. citri
Source: BMC Genomics. 2015 Jul 14;16(1):524. doi: 10.1186/s12864-015-1736-5 (PMC4501297; doi:10.1186/s12864-015-1736-5)
Supplement: Additional file 2: Table A2. — List of proteins and their hosts used for the phylogenetic analyses of the periplasmic components from X. citri belonging to the putatives sulfate and organosulfur compounds pathways. [file 12864_2015_1736_MOESM2_ESM.pdf]

Additional File 4

Microorganism	Code	GI	Protein Function
Acinetobacter baumannii	Abau	479901955	hypothetical protein F983_01342
Agrobacterium tumefaciens C58	Atu	159184478	ABC transporter, substrate binding protein (sulfate)
Azotobacter vinelandii	Avd	226945121	sulfate ABC transporter-binding component-CysP-like
Azotobacter vinelandii	Avd	482537325	ABC transporter, aliphatic sulfonate binding protein
Azotobacter vinelandii	Avd	482900534	ABC transporter, taurine periplasmic binding protein
Bradyrhizobium japonicum USDA110	Bja	27376593	sulfate ABC transporter substrate-binding protein
Burkholderia cenocepacia	Bcm	170732926	sulfate ABC transporter substrate-binding protein
Burkholderia cenocepacia	Bcm	169820876	aliphatic sulfonates family ABC transporter
Cellvibrio japonicus	Cja	192360321	sulfate ABC transporter substrate-binding protein
Chromobacterium violaceum	Cvi	34497287	sulfate transport system sulfate-binding protein
Chromobacterium violaceum	Cvi	34104167	taurine transport system substrate-binding protein
Citrobacter freundii	Cfre	411773071	taurine transporter substrate binding subunit
Cronobacter dublinensis	Cdu	426743387	alkanesulfonates-binding protein
Cronobacter sakazakii	Esa	156533345	hypothetical protein ESA_02942
Enterobacter cloacae subsp. cloacae	Enc	296105385	putative sulfur-binding protein
Enterobacter cloacae subsp. cloacae	Enc	295057506	alkanesulfonate transporter substrate-binding subunit
Enterobacter cloacae subsp.	Enc	296101487	sulfonate/nitrate/taurine transport system substrate-

cloacae			binding protein
Erwinia billingiae	Ebi	15802955	sulfate/thiosulfate transporter subunit
Erwinia billingiae	Ebi	299061943	Aliphatic sulfonates binding protein
Erwinia billingiae	Ebi	300718731	taurine ABC transporter substrate-binding protein
Escherichia coli K-12	Eco	16131755	sulfate transporter subunit
Escherichia coli K-12	Eco	90111189	aliphatic sulfonate binding protein
Escherichia coli K-12	Eco	90111122	taurine transporter subunit
Geobacter sulfurreducens PCA	Gsu	39996448	sulfate ABC transporter substrate-binding protein
Herbaspirillum seropedicae	Hse	300072771	ABC-type nitrate/sulfonate/bicarbonate transport system
Hylemonella gracilis	Hgra	332041366	aliphatic sulfonate ABC transporter periplasmic ligand-binding protein
Janthinobacterium sp. Marseille	Jmar	151281655	ABC-type nitrate/sulfonate/bicarbonate transport systems, periplasmic component
Klebsiella pneumoniae	Kpn	152972514	sulfate transporter subunit
Klebsiella pneumoniae	Kpn	471247537	aliphatic sulfonate ABC transporter substrate-binding protein
Klebsiella pneumoniae	Kpn	480517614	taurine ABC transporter substrate-binding protein
Mesorhizobium loti	Mlo	13471636	sulfate binding protein of ABC transporter
Methylobacillus flagellatus KT	Mfla	91775916	sulfonate ABC transporter, periplasmic sulfonate-binding protein SsuA
Methylobacillus flagellatus KT	Mfla	91775917	sulfonate ABC transporter, periplasmic sulfonate-binding protein SsuA
Methyloversatilis universalis FAM5	Mun	333444678	ABC-type nitrate/sulfonate/bicarbonate transport system
Minibacterium massiliensis	Mms	152980434	sulfate ABC transporter substrate-binding protein

(Janthinobacterium sp. Marseille)			
Mycobacterium tuberculosis H37Rv	Mtu	15609537	probable sulfate-binding lipoprotein SubI
Pantoea sp. GM01	Pant	398100345	ABC transporter, substrate-binding protein, aliphatic sulfonates family
Pantoea sp. GM01	Pant	398092540	taurine ABC transporter, periplasmic binding protein
Pectobacterium atrosepticum	Eca	50122221	sulfate-binding protein
Pectobacterium sp. SCC3193	Pec	385869651	aliphatic sulfonates family ABC transporter, periplasmic ligand-binding protein
Polaromonas naphthalenivorans	Pna	120592425	aliphatic sulfonates family ABC transporter, periplasmic ligand-binding protein
Pseudomonas aeruginosa	Pae	15595480	sulfate-binding protein
Pseudomonas aeruginosa	Pae	428157993	putative sulfonate binding protein
Pseudomonas aeruginosa	Pae	428155815	alkanesulfonate transporter substrate-binding subunit
Pseudomonas aeruginosa	Pae	497907063	taurine ABC transporter substrate-binding protein
Ralstonia solanacearum	Rso	17546055	sulfate-binding signal peptide protein
Ralstonia solanacearum	Rso	299067394	alkanesulfonates binding signal peptide protein
Rhodopseudomonas palustris CGA009	Rpa	39933827	thiosulfate-binding protein
Rubrivivax gelatinosus	Rge	381379056	taurine ABC transporter substrate binding protein TauA
Salmonella enterica subsp. enterica serovar Typhimurium LT2	Stm	16767329	sulfate transporter subunit
Serratia proteamaculans	Spe	157321745	aliphatic sulfonates family ABC transporter, periplsmic ligand-binding protein

Thauera sp. 27	Tmz	479290628	nitrate/sulfonate/bicarbonate ABC transporter periplasmic protein
Xanthomonas axonopodis pv. citri 306	Xac	21241782	sulfate ABC transporter substrate-binding protein
Xanthomonas axonopodis pv. citri 306	Xac	21241619	sulfonate-binding protein
Xanthomonas axonopodis pv. citri 306	Xac	21243924	alkanesulfonate transporter substrate-binding subunit
Xanthomonas axonopodis pv. malvacearum	Xama	410707920	sulfonate-binding protein
Xanthomonas axonopodis pv. punicae	Xapu	372555421	ABC transporter, substrate-binding , aliphatic sulfonates family protein
Xanthomonas axonopodis pv. punicae	Xapu	372555953	nitrate transport protein
Xanthomonas campestris pv. campestris	Xcc	21230409	sulfate ABC transporter substrate-binding protein
Xanthomonas campestris pv. campestris	Xcc	498049206	alkanesulfonate transporter substrate-binding subunit
Xanthomonas citri pv. mangiferaeindicae	Xcma	380686748	nitrate transport protein
Xanthomonas fuscans subsp. Aurantifolii	Xfau	292599603	sulfonate binding protein
Xanthomonas fuscans subsp. Aurantifolii	Xfau	292603243	nitrate transport protein
Xanthomonas gardneri	Xga	325547110	ABC transporter, substrate-binding protein, aliphatic sulfonates family

Xanthomonas gardneri	Xga	325546958	ABC transporter, substrate-binding protein, aliphatic sulfonates family
Xylella fastidiosa Temecula1	Xft	28198498	sulfate ABC transporter substrate-binding protein
Yersinia pestis biovar Antiqua	Ypa	167051183	taurine ABC transporter, periplasmic taurine-binding protein
Yersinia pestis CO92 (biovar Orientalis)	Ype	488142647	sulfonate ABC transporter substrate-binding protein
