# Supplementary material for: The sulfur/sulfonates transport systems in Xanthomonas citri pv. citri
Source: BMC Genomics. 2015 Jul 14;16(1):524. doi: 10.1186/s12864-015-1736-5 (PMC4501297; doi:10.1186/s12864-015-1736-5)
Supplement: Additional file 3: Table A3. — Microorganisms and access code for the 16S rRNA sequences used to show the conservation of the putatives sulfate and organosulfur compounds pathways proteins in different classes. [file 12864_2015_1736_MOESM3_ESM.pdf]

Additional File 5

Microorganism	Code	Kegg entry or G.I. 16S rRNA sequences
Agrobacterium tumefasciens	Atu	Atu0053
Bacillus subtilis subsp. subtilis 168	Bsu	BSU_rRNA_1
Bradyrhizobium japonicum USDA110	Bja	Bjar01
Burkholderia cenocepacia MC0-3	Bcm	Bcenmc03_R0017
Cellvibrio japonicus	Cja	CJA_3859
Chromobacterium violaceum	Cvi	CV_rRNA16s1
Citrobacter rodentium	Cro	16S_rRNA-4
Enterobacter cloacae subsp. cloacae	Enc	ECL_16S03
Erwinia billingiae	Ebi	EbC_08400_tr01
Escherichia coli K-12 MG1655	Eco	b0201
Geobacillus thermodenitrificans	Gtn	GTNG_16s004
Geobacter sulfurreducens PCA	Gsu	GSUR010
Herbaspirillum seropedicae	Hse	Hsero_0477
Klebsiella pneumoniae subsp. pneumoniae	Kpn	KPN_03679
Mesorhizobium loti	Mlo	MAFFr03
Methanosarcina mazei Go1	Mma	MM_ZRNA060
Methyloversatilis universalis FAM5	Mun	G.I.:343198836
Pectobacterium atrosepticum	Eca	ECAr004
Pseudomonas aeruginosa PAO1	Pae	PA0668.1
Ralstonia solanacearum CMR15	Rso	RS05422
Rhodopseudomonas palustris CGA009	Rpa	RPA_RNA57
Salmonella enterica subsp. enterica serovar Typhimurium LT2	Stm	STM0249
Shigella flexneri 301 (serotype 2a)	Sfl	SF4435
Xanthomonas axonopodis pv. citri 306	Xac	XAC3896
Xanthomonas campestris pv. campestris ATCC 33913	Xcc	XCC3840
Xanthomonas campestris pv. vesicatoria	Xcv	XCVr2
Xanthomonas oryzae pv. oryzae KACC 10331	Xoo	XOO4692
Xylella fastidiosa Temecula1	Xft	PD0048
