# Supplementary material for: The sulfur/sulfonates transport systems in Xanthomonas citri pv. citri
Source: BMC Genomics. 2015 Jul 14;16(1):524. doi: 10.1186/s12864-015-1736-5 (PMC4501297; doi:10.1186/s12864-015-1736-5)
Supplement: Additional file 4: Table A4. — Proteins with three-dimentional structure solved used as template for the molecular modeling of the putative sulfur pathway components identified in X. citri. Amino acid sequence alignments were performed using ClustalW [35]. [file 12864_2015_1736_MOESM4_ESM.pdf]

Additional File 6

**Protein/ *X. citri***	**PDB Template**	**Function**	**Aminoacid Sequence (%) Identity /Coverage**	**Reference**
SsuA(1)	3E4R, Chain A	Alkanesulfonate-binding protein SsuA from *Xanthomonas citri*	22/98	[19]
SsuD(1)	1M41, Chain A	Alkanesulfonate monooxygenase SsuD from *Escherichia coli*	64/94	[28]
SflA	4PTZ, Chain D	Alkanesulfonate FMN reductase SsuE from *Escherichia coli*	26/83	[29]
