# Supplementary material for: The sulfur/sulfonates transport systems in Xanthomonas citri pv. citri
Source: BMC Genomics. 2015 Jul 14;16(1):524. doi: 10.1186/s12864-015-1736-5 (PMC4501297; doi:10.1186/s12864-015-1736-5)
Supplement: Additional file 5: Table A5. — Oligonucleotides used for gene amplification in the RT-PCR analysis performed in this study. F: forward and R: reverse oligonucleotides. PCR reactions were described in Methods, using the annealing temperature of 51 °C for all reactions. [file 12864_2015_1736_MOESM5_ESM.pdf]

Additional File 7

Gene/KEGG Reference	Orientation/ Restiction enzyme	Nucleotide sequence
sbp/Xac1017	F/*Bam*HI	5´ GGTGGATCCTTGCTGGTCC 3´
	R/*Hind*III	5´ AAGCTTTTACTTGTTCGGCTG 3´
cysU/Xac1018	F/*Eco*RI	5' CAAGCTTTCATACGGGCC 3’
	R/*Hind*III	5' GGAATTCTATGTCCATGTCG 3’
cysW/Xac1019	F/*Bam*HI	5' GGGATCCAATGAACGATGC 3’
	R/*Hind*III	5' GAAGCTTTCAATGGCGGTC 3’
cysA/Xac1020	F/*Bgl*II	5' GAAGATCTAGTGACATGG 3’
	R/*Xho*I	5' GGCTCGAGTCACGCGGG 3’
cysD/Xac3329	F/*Nde*I	5' CATATGACTCTGCCGCC 3'
	R/*Eco*RI	5' GAATTCTCAGAAATACCC C 3'
cysNC/Xac3328	F/*Nde*I	5' CATATG GGCAGCGAATGG 3'
	R/*Bam*HI	5' GGATCCTTAGCGTTGCAG 3'
cysJ/Xac3330	F/*Nde*I	5' CATATGACCGCCGCCAGT 3’
	R/*Bam*HI	5' GGATCCCTAATAGACATCTC 3’
cysI/Xac3331	F/*Nde*I	5' CATATGAGCCACTCCGTC 3’
	R/*Xho*I	5’ CTCGAGTCATGCGAGCAATTCCAGGTCG 3’
cysH/Xac3332	F/*Nde*I	5’ CATATGACCGCGCTGCCTGCTGCA 3’
	R/*Xho*I	5’ CTCGAGCTAGATATCTTCGTGGATGCCG 3’
ssuA1/Xac0849	F/*Nde*I	5’ CATATGAAGCGTCGCAACCTGCTCA 3´
	R/*Xho*I	5’ CTCGAGTCAGGCCCGCGCCACGCCTG 3´
ssuB1/Xac0847	F/*Nde*I	5’ CATATGGTGAGCAGCTCCGGCCTGCAG 3’
	R/*Xho*I	5’ CTCGAGTCAGAACACATGGAGGCTGTGC 3’

ssuC1/Xac0848	F/*Nde*I	5’ CATATGAACGACACCTCCTTGC 3’
	R/*Xho*I	5’ CTCGAGTCACCTAGGGCTCCCAGGGTG 3’
ssuD1/Xac0850	F/*Nde*I	5’ CATATGGACATGTTCTGGTTCATCCC 3'
	R/*Xho*I	5’ CTCGAGTCAGCGCGCCGAAGCGCGCG 3'
slfA/Xac0851	F/*Nde*I	5' CATATGCGCAGTCCGTTGAAT 3'
	R/*Xho*I	5' CGCAACAACGCATCGGT 3'
ssuA2/Xac3198	F/*Nde*I	5’ CATATGCGGGCAACGGGCAGGCAG 3’
	R/*Sal*I	5’ GTCGACTCATTTGCTCACCGCCTGCGCT 3’
ssuB2/Xac3196	F/*Nde*I	5’ CATATGGTGGCTGGGCTGGCATCCGGC 3’
	R/*Xho*I	5’ CTCGAGCTAGAGGATGCGTTGCAACGGC 3’
ssuC2/Xac3197	F/*Bam*HI	5' GGGATCCAATGAGCGCCG 3’
	R/*Hind*III	5' CAAGCTTTCATCGGCGTAGC 3’
ssuD2/Xac3199	F/*Nde*I	5’ CATATGCGCTATCTCGCACATCCCG 3’
	R/*Xho*I	5’ CTCGAGTCATTGCCAATCGGTGGACGGC 3’
ssuE2/Xac3200	F/*Nde*I	5’CATATGAGCGCGCCACGTCAGCTG 3’
	R/*Hind*III	5’ CTCGAGTCAGCGTGCGCTGAAGCGATTG 3’
cysB/Xac3339	F/*Nde*I	5' GCATATGACGCTGACCCAA C 3'
	R/*Bam*HI	5' GGATCCTCAGCTGGTGATG 3'
cysG/Xac3340	F/*Nde*I	5' CATATGAAGTCATTTCCGACGG 3’
	R/*Bam*HI	5’ GGATCCTCAGGCTGCGTG 3’
16S rRNA/XAC3896	16S_F	5’ TGGTAGTCCACGCCCTAAACG 3’
	16S_R	5’ CTGGAAAGTTCCGTGGATGTC 3’
