# Supplementary figures and images for: The sulfur/sulfonates transport systems in Xanthomonas citri pv. citri
Source: BMC Genomics. 2015 Jul 14;16(1):524. doi: 10.1186/s12864-015-1736-5 (PMC4501297; doi:10.1186/s12864-015-1736-5)

**A**

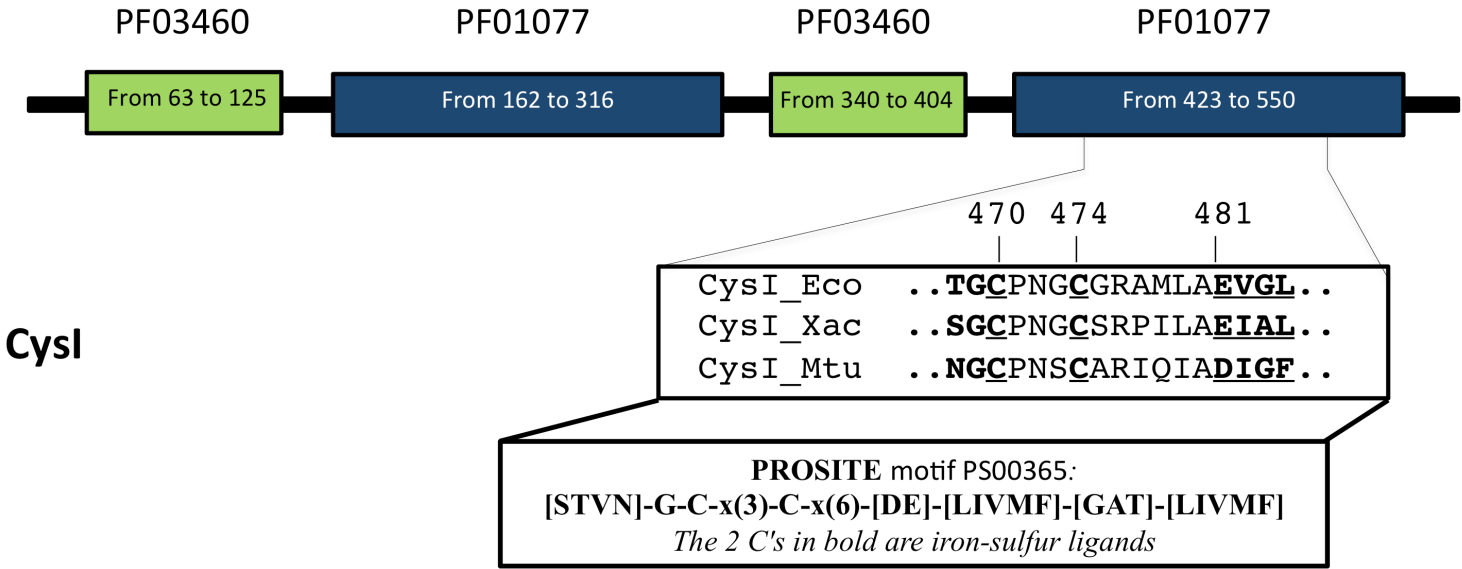

**B**

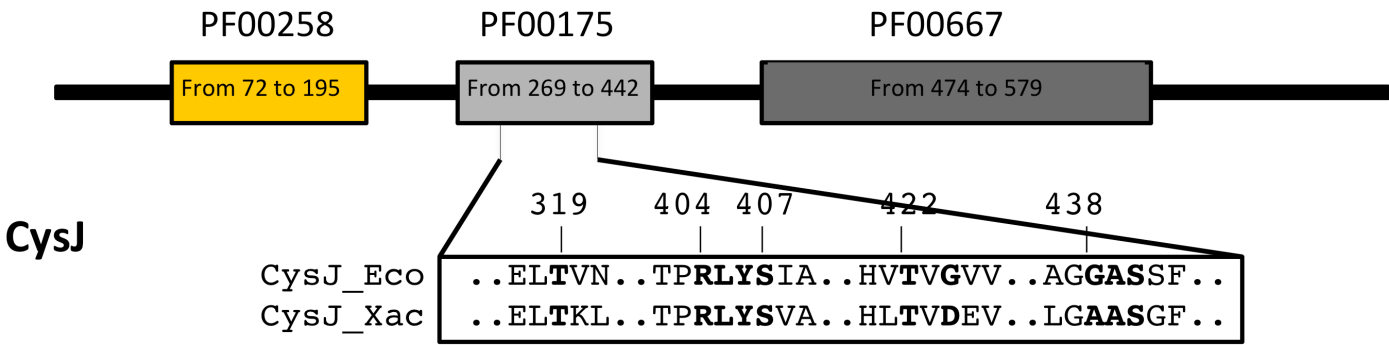

Supplement: Additional file 6: Figure A1. — Domain structure of the putative CysI and CysJ of X. citri. (A) PFAM domain structure of the putative CysI of X. citri. PF03460 = nitrite/sulfite reductase ferrodoxin-like half domains and PF01077 = nitrite and sulfite reductase 4Fe-4S binding and siroheme binding. The amino acid sequence (residue numbers 468 to 484) of the PF01077 domain corresponding to the C-terminus of the 4Fe-4S binding and siroheme binding domain is aligned with similar regions of CysI from Escherichia coli (KEGG entry: b2763) and Mycobacterium tuberculosis H37Rv (gi: 15609528). Conserved amino acids are underlined bold. The sequence of the Prosite motif PS00365, [STV]-G-C-x(3)-C-x(6)-[DE]-[LIVMF]-[GAT]-LIVMF] is also shown. (B) PFAM domain structure of the putative CysJ of X. citri. PF00258 = flavodoxin domain, PF00667 = FAD binding domain and PF00175 = NAD-binding domain. The amino acid sequence (residue numbers 317 to 442) of the PF00175 domain is aligned with similar regions of CysJ from Escherichia coli (KEGG entry: b2764). [file 12864_2015_1736_MOESM6_ESM.pdf]

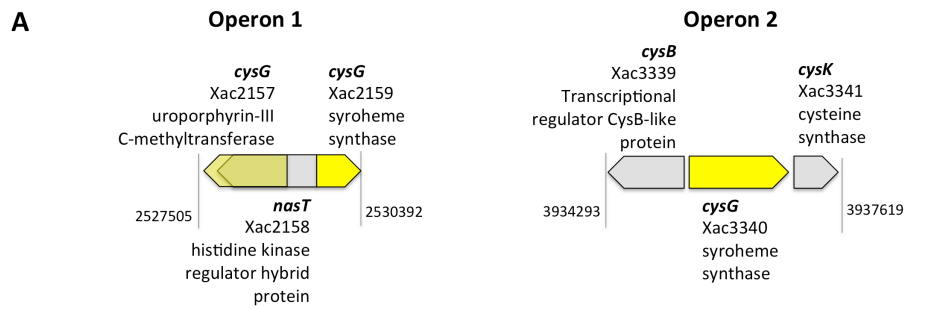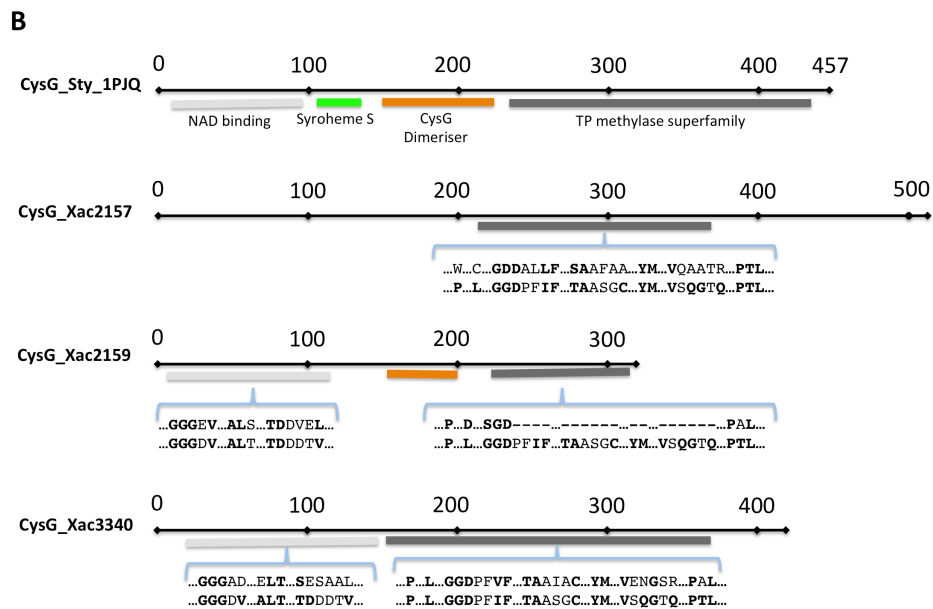

Supplement: Additional file 7: Figure A2. — Comparison of the three putative CysG protein sequences found in X. citri with the siroheme synthase CysG from S. typhimurium. (A) Positioning of the cysG genes in the X. citri genome. Xac2159 and Xac2157 genes, encoding respectively, an uroporphyrin-III C-methyl transferase and a syroheme synthase, are closely located in opposite directions. The third gene, Xac3341, encoding another syroheme synthase is located in the same predicted operon with the cysK gene that encodes a cysteine synthase. (B) Putative conserved domains identified in the three CysG amino acid sequences in comparison with the multifunctional methyltransferase/dehydrogenase/ferrochelatase CysG from S. typhimurium, which three-dimensional structure was solved in presence of S-adenosyl-L-homocysteine (SAH) [1]. The amino acid sequence alignment from the identified domains is shown evidencing the conservation of the residues involved with SAH and NAD interactions (showed in bold). [file 12864_2015_1736_MOESM7_ESM.pdf]
